# 3D CT-Based Preoperative Planning and Intraoperative Navigation in Reverse Shoulder Arthroplasty: Early Clinical Outcomes

**DOI:** 10.3390/medicina61040749

**Published:** 2025-04-18

**Authors:** Elisa Troiano, Azzurra Masini, Giovanni Battista Colasanti, Caterina Drago, Stefano Giannotti, Nicola Mondanelli

**Affiliations:** 1 Department of Medicine, Surgery and Neurosciences, University of Siena, Viale Mario Bracci 16, 53100 Siena, Italy; 2 Section of Orthopedics, Azienda Ospedaliero Universitaria Senese, Policlinico Santa Maria alle Scotte, Viale Mario Bracci 16, 53100 Siena, Italy

**Keywords:** reverse shoulder arthroplasty, preoperative planning, intraoperative navigation, computer-assisted surgery, radiographic outcomes, clinical outcomes, level of evidence, level IV, retrospective study

## Abstract

*Background and Objectives*: Reverse shoulder arthroplasty (RSA) is an effective surgical procedure for treating end-stage rotator cuff arthropathy, but it is burdened by a relatively high complication rate, mainly due to glenoid component failure. Preoperative planning and intraoperative navigation based on three-dimensional computed tomography (3D CT) scans have proven to be efficient tools for improving the accuracy and stability of the glenoid component. However, this technology is still developing, and there is currently little available research on the subject, especially where clinical outcomes are concerned. The purpose of this retrospective observational study is to report the radiographic and clinical outcomes of a consecutive series of patients that underwent RSA with the use of these new technologies, compared to a standard procedure. *Materials and Methods*: A consecutive series of 80 patients underwent RSA for shoulder osteoarthritis by a single surgeon at a single institution with a mean follow-up of 41.9 ± 23.6 months (range 24–108) and were divided into two groups according to the surgical technique employed (conventional or navigated surgery), and they were clinically and radiographically assessed at 1, 3, 6, and 12 months after surgery, and then annually. *Results*: No statistically significant differences were highlighted among the two groups according to complication rate, radiographical glenoid notching, and clinical outcomes. However, a statistically significant difference was observed in screw number and length and surgical time. In the navigated group, fewer screws with longer lengths had been implanted, with a longer surgical time. *Conclusions*: The use of 3D CT-based preoperative planning and intraoperative navigation is a safe procedure and produces comparable results with respect to standard instrumentation, without an increased risk of complications. It allowed to achieve higher stability of the implant, saving bone stock due to the use of fewer and longer screws than in a conventional procedure. This could also eventually result in a higher longevity of the implant itself.

## 1. Introduction

The demand for reverse shoulder arthroplasty (RSA) is rising, accompanied by the imperative to minimize risks and complications related to implant placement. Optimal positioning of components, especially the glenoid, poses considerable challenges due to factors such as limited surgical exposure, glenoid deformities, and bone deficiencies [1,2]. Complex glenoid bone loss and deformity remain significant challenges for shoulder arthroplasty surgeons in both primary and revision procedures. These conditions reduce the already limited available surface area and bony support for glenoid component implantation, increasing the risk of early loosening and, ultimately, implant failure [3]. Improper positioning of the glenoid may result in significant complications, including implant loosening, scapular notching, impingement, and instability, eventually leading to revision surgery [2,4,5]. To address these challenges, advanced imaging techniques, planning software, patient-specific instruments, and computer-assisted navigation have been employed to improve the accuracy of glenoid placement. These technologies provide surgeons with detailed intraoperative guidance, improving the accuracy of instrument and implant positioning while offering real-time feedback on screw placement and baseplate fixation [1,5,6].

The adoption of computer-assistance has progressively increased in recent years, reflecting its growing integration into clinical practice [7] and extending also to complex situations such as revision surgery [8,9]. However, despite this trend, the specific benefits of intraoperative computer-assisted navigation for shoulder prosthetic procedures remain debatable, with respect to conventional surgery. Some studies have reported advantages such as enhanced component alignment and a lower risk of complications, yet definitive evidence regarding its overall efficacy in shoulder arthroplasty is still lacking [4,10]. This uncertainty highlights the need for further research to evaluate the true impact of these advanced technologies on the outcomes of shoulder prosthetic implantation.

The aim of the present study is to assess the use of preoperative planning and intraoperative navigation compared to a conventionally planned technique, emphasizing the benefits of using fewer but longer screws. This study originates from our previous one from 2020 [11]. The sample size has been expanded, and new variables have been considered, particularly those related to clinical and radiographic outcomes. We hope that our findings may contribute to the existing literature on the advantages of navigation in RSA, providing further insights into its clinical utility.

## 2. Materials and Methods

*Study design.* A retrospective and observational study was conducted, analyzing all RSA cases performed at our institution up to December 2022. Patients were included based on the following criteria: (1) diagnosis of eccentric shoulder osteoarthritis or rotator cuff arthropathy and who had a contralateral healthy shoulder, (2) treatment with RSA using three-dimensional computed tomography (3D CT)-based preoperative planning (Exactech Guided Personalized Surgery software 2.1 version; ExactechGPS®, BlueOrtho, Gières, France), (3) surgery performed by the same surgeon (S.G.), (4) the use of a single specific prosthetic implant (Equinoxe^®^ Reverse System; Exactech, Gainesville, FL, USA), and (5) a minimum follow-up period of 24 months. Patients were excluded if they had sustained previous scapular or humeral fractures, if the RSA was performed for proximal humeral fracture, as a revision procedure (for failed internal fixation of the proximal humerus, or failed previous prosthetic implant), or if a custom implant was needed. Also, patients with contralateral surgical shoulder procedures were excluded. Patients were divided into two groups based on the type of procedure: NAV, for procedures conducted with the assistance of intraoperative navigation after preoperative planning, and CONV, for procedures performed using the conventional approach when intraoperative navigation was not possible after preoperative planning was done. No allocation criteria based on the complexity of the glenoid defect were applied to either group, ensuring the avoidance of selection bias.

Until 2018, the year intraoperative navigation technology was introduced at our center, all patients underwent conventional procedures. After this date, conventional procedures were reserved for cases where coracoid damage was severe enough to preclude secure fixation of the scapular tracker without causing further harm to the patient or when planning validation was not available by the date of surgery.

At our institution, no Ethical Committee approval is necessary for retrospective studies, and all patients gave their informed consent to data collection and their anonymous use for scientific purposes.

**Figure 1:**
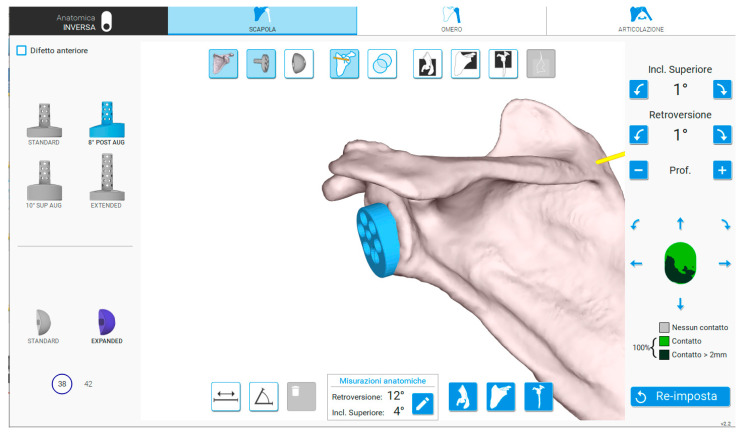
Interface of the software enabling planning with 3D CT reconstructions. On the left side of the image, the glenoid components (baseplate and glenosphere) can be selected and positioned on the 3D model. On the right side, the degrees of inclination and retroversion to be applied to the baseplate, as well as the percentage of baseplate contact with the native bone, are displayed. Additionally, it is possible to overlay the CT slices onto the 3D reconstruction.

*Preoperative phase.* All patients underwent preoperative CT scan imaging of the shoulder, meeting the specifications required by the planning software [11]. Following acquisition, the CT scan was uploaded to the online planning software, which automatically anonymized patient data. Subsequently, after identifying specific anatomical landmarks, the system generated a 3D model of the scapula. Using this model, the surgeon planned the implant, selecting the type of baseplate and glenosphere and determining final implant positioning (Figure 1). 

To use the intraoperative navigation system, the preoperative plan must be validated by the engineering team. Once validation is sent back, the surgeon can revise it if necessary, and subsequently uploads it to the intraoperative application device.

*Surgical procedure.* All surgeries were performed by the same senior surgeon with extensive experience in RSA procedures for both traumatic and chronic degenerative conditions. In all cases, the same prosthetic implant was utilized. The following describes the surgical procedure aided by intraoperative navigation.

**Figure 2:**
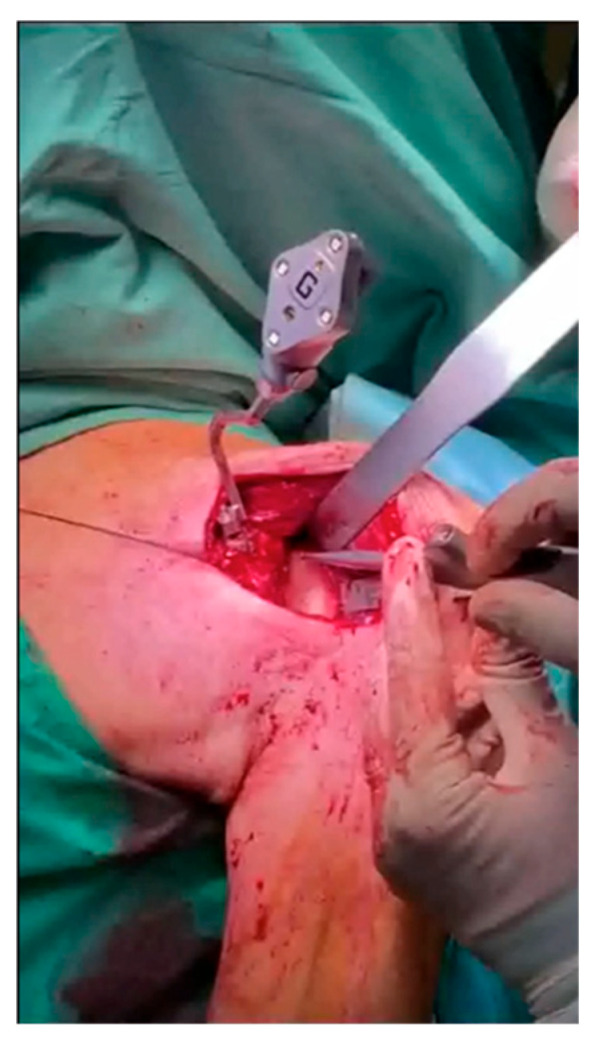
Intraoperative acquisition of scapular bony landmarks with a handheld tracker.

A computerized interface with a touchscreen monitor was included within the surgical field. A deltopectoral approach, extended proximally by approximately 1.5 cm, was used in all cases to ensure better visualization of the coracoid process. After skin incision and subscapularis tenotomy, the humeral side was prepared using conventional instrumentation. At this stage, the superior surface of the coracoid process was prepared for the placement of the scapular tracker, which was secured with two screws, ensuring it remained stable throughout the entire procedure. Next, the glenoid was exposed, carefully removing soft tissues as needed. Intraoperative markers were captured using a handheld tracker (Figure 2), mapping key anatomical landmarks (anterior and posterior surface and baseline of the coracoid process; superior, inferior, anterior, and posterior margins of the glenoid surface; anterior, inferior, and posterior rim lines of the glenoid; anterior and inferior neck lines). This allowed verification and overlay of the 3D model with the patient’s anatomy.

**Figure 3:**
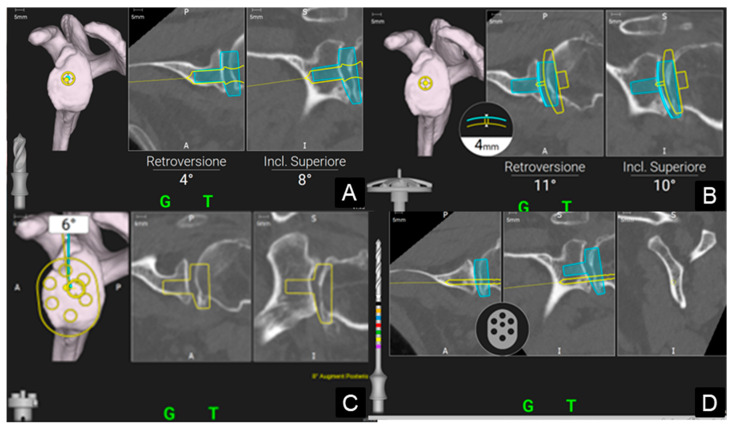
Stages of intraoperative navigation as displayed on the monitor within the sterile field. Real-time feedback is provided regarding the progression of the instrument within the patient’s bone, allowing simultaneous trajectory corrections. (**A**) Navigation of the central peg, (**B**) navigation of glenoid reaming, (**C**) navigation of baseplate positioning and (**D**) navigation of screws.

Once acquisition was complete, the preoperative plan was displayed on the monitor, enabling real-time navigation during each step of the procedure: pilot hole creation and positioning verification, glenoid reaming, baseplate positioning, and screw navigation (Figure 3). Screw lengths were displayed both on the monitor and via a color-coded system on the drill bit. 

After screw placement, intraoperative navigation concluded with the removal of the coracoid tracker. The surgical procedure then proceeded conventionally with glenosphere placement, definitive humeral implant insertion, and subscapularis repair. To minimize bleeding, tranexamic acid was administered both intravenously and intra-articularly in the absence of contraindications, as previously described [12]. A drain was left in place for 24 h. 

*Postoperative care.* All patients followed the same postoperative protocol to minimize variations in functional outcomes attributable to differences in rehabilitation protocols. The arm was immobilized in a 45° abduction brace for 3 weeks, allowing passive mobilization of the shoulder while avoiding the maximum degrees of rotational movements. This approach reduced tension on the subscapularis and enhanced prosthetic stability. Active and passive mobilization of the elbow and the wrist was permitted during this period. Upon brace removal at 3 weeks, physiotherapy was initiated. Active mobilization exercises in anterior elevation and abduction were encouraged at 3 weeks. Rotational movements and muscle-strengthening exercises began at 5 weeks postoperatively. Lifting heavy objects was restricted until at least 9 weeks post-surgery, with patients returning to full activities at 3 months postoperatively.

**Figure 4:**
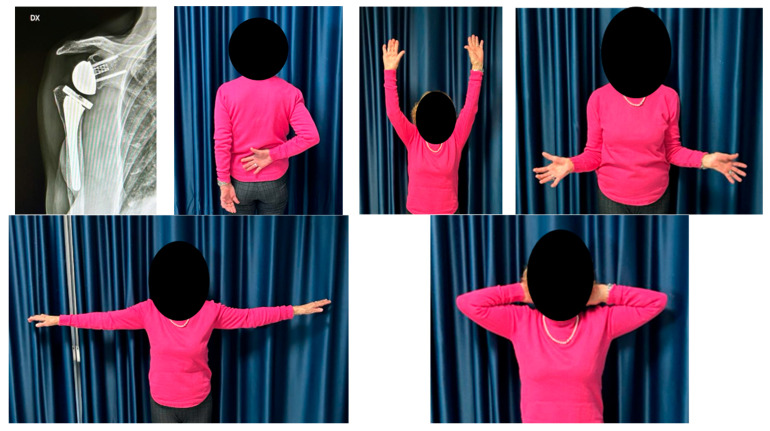
Clinical and radiographical outcomes of a 76-year-old woman at a 3-year follow-up for the right shoulder (NAV group).

*Clinical and radiological assessment.* All patients underwent clinical and radiographic evaluation at 1, 3, 6 and 12 months postoperatively, and then annually (Figure 4). Shoulder function was assessed using the Constant score [13], analyzing both absolute values and differences relative to the contralateral healthy limb.

Given that the Constant score (where 0 points is the minimum and 100 points is the maximum) is not highly accurate in evaluating strength and may fail to provide a comprehensive assessment of functional outcomes, some authors recommend comparison against a homogeneous control group matched for sex and age [14] or the use of the contralateral limb for personalized benchmarking [15]. Patient-reported outcomes were further assessed using the Disability of the Arm, Shoulder and Hand (DASH) score [16], where 0 points is the maximum and 100 points is the worst results.

Radiographs in the true anteroposterior (Grashey, [17]) projection were obtained at each visit. Glenoid notching was evaluated according to the Nerot-Sirveaux classification [18].

*Statistical analysis.* Quantitative variables’ normal distribution was verified using the Shapiro–Wilk test. However, since the data did not follow a normal distribution, statistical analysis was performed using non-parametric tests for both continuous and categorical variables. Specifically, statistical analysis was performed using the Mann–Whitney test for continuous variables and the chi-square test for categorical variables, with Yates correction applied when the total number of observations ranged from 40 to 200, or Fisher’s exact test for fewer than 40 observations. The significance threshold was set at *p* < 0.05, with non-significant results reported as “n.s.” in the text, and with exact *p*-values in tables. All data were processed using a free online software tool (https://www.socscistatistics.com/, last accessed on 30 November 2024).

## 3. Results

A consecutive series of 80 patients met the inclusion criteria: 22 males and 58 females with a mean age of 74 ± a standard deviation (SD) of 5.7 (range 58–84) years, evaluated at a mean follow-up of 41.9 ± 23.6 (range 24–72) months. Results were reported at the last follow-up visit available. The mean Constant score was 67 ± 16 (range 25–91) points, with a mean Constant score difference of 10 ± 10 (range 0–50) points between the two shoulders. The mean DASH score was 20 ± 19 (range 0.8–84.1) points.

Differences between the NAV and the CONV groups in terms of demographic characteristics, implant features (augmented baseplates, number of screws, and screw length), functional outcomes, and complications are detailed in Table 1. 

The two groups were comparable in age and sex distribution (*p* = n.s.); however, the CONV group had a significantly longer mean follow-up period (*p* = 0.002). No statistically significant differences were observed in terms of the need for transfusions, revisions, functional outcomes (Constant score, DASH score), or complication rates. With respect to complications, five cases were observed in the NAV group: grade-1 glenoid notching (one case), an intraoperative humeral fracture that did not require change in the choice of the stem (one case), acromial fracture (one case), mesoacromial fracture (one case), and late periprosthetic joint infection (PJI) (one case). Three of them (the PJI and the two acromial/mesacromial fractures) required revision surgery. Considering the CONV group, three cases of complications were observed, all of which required revision surgery: late PJI (one case), dislocation of the implant (one case), and breakage of the humeral adapter tray component (one case). No neurovascular injuries were observed in both groups. Similarly, the use of augmented baseplates did not differ significantly between the two groups (*p* = n.s.). However, the NAV group used significantly fewer screws (*p* = 0.0047) of greater length (*p* < 0.00005) compared to the CONV group. Moreover, NAV procedures required, on average, a 7-min longer duration than CONV procedures; however, this difference was not statistically significant (*p* = n.s.).

## 4. Discussion

RSA is a well-established surgical technique for managing end-stage rotator cuff arthropathy. Over the years, its indications have broadened to include cases such as eccentric shoulder osteoarthritis with severe glenoid deformity [19,20,21], complex proximal humeral fractures [22,23,24,25,26], oncologic conditions [27], and revision shoulder arthroplasty with substantial bone deficiency [28]. Despite its success, RSA is associated with a relatively high complication rate, ranging from 19% to 68% [29], including hematoma, neurological injury, periprosthetic fracture, PJI, instability, dislocation, scapular notching, impingement, acromial fractures, and mechanical baseplate failure [1,28]. Among these, glenoid component failure is one of the most frequent postoperative complications, and improper glenoid positioning has been shown to contribute to humeral instability, increased stress at the bone-prosthesis interface, premature failure, and poor postoperative outcomes [30]. Consequently, achieving precise positioning and stable fixation of the baseplate is critical for implant stability, postoperative function, and long-term survival [2,11,31,32,33]. However, these goals are technically demanding due to limited visibility, poorly defined bony landmarks, and the complex morphology of the glenoid, especially in pathological settings.

As a result, proper preoperative planning and intraoperative tools that assist surgeons in minimizing errors are of paramount importance, particularly in shoulder surgery. Three-dimensional CT-based preoperative planning combined with intraoperative navigation systems has been increasingly adopted to address these challenges. These technologies improve the accuracy of component placement, similar to their use in hip and knee arthroplasty, enhance implant stability and conserve bone stock by allowing the use of fewer but longer screws. Such advantages have been supported in the existing literature [1,4,11,34]. It has been demonstrated that a higher number of screws can reduce the bone stock available for baseplate fixation [35]. This concept, along with the physiological anatomical characteristics of the glenoid, is particularly crucial when considering the need of potential implant revisions. The native glenoid bone stock is highly limited and preserving it may allow for implant revision using a less complex system, decreasing complications for patients as a consequence.

This study confirms that patients undergoing surgery with 3D CT-based planning and intraoperative navigation (NAV group) required fewer but longer screws for fixation compared to the CONV group. These findings align with the current literature [1,4,11,34,36,37], particularly with a recent systematic review and meta-analysis [38], which reported comparable results across six clinical studies. However, some differences regarding the choice of baseplates emerge. This tool allowed the surgeon to better understand the patient’s anatomy and choose the most appropriate baseplate to address bone defects [36]. According to the present findings, there were no differences between the groups regarding the use of augmented baseplates, as some authors previously described [4]. In other cases, opposite results were observed [1,11,36,37,39,40]. This difference can be explained by the fact that all procedures were planned using 3D CT-based preoperative planning, and by the lack of a control group for variable planning. 

Regarding surgical time, the NAV group exhibited an average increase of 7 min per procedure. This difference can be attributed to the time required for image acquisition and alignment between the 3D CT-based model and the patient’s anatomy. Nevertheless, this increase in operative time was neither statistically nor clinically significant [4,40], unlike what some studies have highlighted [34,39].

Given the novelty of intraoperative navigation technology, the current body of literature is limited and primarily reports short-term results in small patient cohorts. The present study adds to this body of work, confirming that no significant differences exist in objective or subjective outcomes between the NAV and CONV groups. Both Constant scores and DASH scores were comparable across groups, consistent with prior studies [1,4,37]. However, interpreting these results in isolation is challenging. When analyzing the absolute Constant score, or the difference in Constant score between the affected and contralateral shoulders, different insights into the quality of outcomes emerge. The mean Constant score of 67 points suggests a moderate outcome. However, it is well-documented that the Constant score inadequately evaluates shoulder strength and is influenced by factors such as patient age and sex. A more accurate assessment could involve comparing patients to a healthy population with similar demographics [14] or simply evaluating the contralateral shoulder [15]. In the present study, the mean difference in Constant score between the affected and contralateral shoulders was approximately 10 points in both groups, corresponding to an excellent outcome. Additionally, in 59% of patients, clinical outcomes were comparable to the contralateral side, with 40% of these cases showing better results than the unaffected shoulder. This underscores the limitations of using the Constant score as an absolute value to gauge real patient outcomes.

The rates of complications and revisions were comparable between the two groups in the present study, even if some evidence to the contrary is present in the literature [1]. In the NAV group, the complication rate was 9%, including grade-1 scapular notching (1.8%), PJI (1.8%), intraoperative humeral fractures (1.8%), and acromial (1.8%) and mesoacromial fractures (1.8%). The revision rate in this group was 5.6%.

In the CONV group, the complication rate was slightly higher at 11%, with a similar revision rate, as all complications necessitated reoperation. Specifically, complications included PJI (3.7%), implant dislocation (3.7%), and humeral tray fractures (3.7%). These findings are consistent with prior studies [1,5,37]. Moreover, no neurovascular injury was observed in both groups. This finding is particularly important as it highlights the safety of new technologies, especially intraoperative navigation, which enables the placement of longer screws while minimizing the risk of damage to neurovascular structures.

No significant differences were observed in transfusion rates between the two groups (7.5% vs. 7.4%, respectively), likely due to a standardized intraoperative protocol for blood loss management applied consistently across all patients without contraindications.

The present study has several strengths but also some limitations. All procedures were performed by a single experienced surgeon, minimizing operator-related variability and reducing the influence of the learning curve typically associated with navigation systems on surgical times. This may be considered a limitation, but in small cohorts such as the present one, it may be considered a strength, reducing possible surgeon-related biases. Furthermore, patients were not preselected based on the complexity of their glenoid bone loss, limiting bias in postoperative outcomes related to bone defect severity.

The sample size of 80 patients is reasonably representative; however, the uneven distribution between the NAV and CONV groups could limit the generalizability of the findings. Additionally, there was a significant difference in follow-up duration, with the CONV group having a longer follow-up period. This could account for the higher complication rate in the CONV group, although the difference was not statistically significant. Similarly, the NAV group follow-up is considered short-term, meaning some complications, such as component loosening, may not have developed yet. Considering that longer screws provide a more stable fixation of the baseplate, the hypothesis is that, in the long term, the revision rate trend will remain stable and lower compared to procedures performed with conventional techniques. However, this hypothesis will need to be validated through long-term studies.

Lastly, the retrospective nature of the study is another limitation. While actual findings align with the existing literature, further long-term and prospective studies are needed to fully elucidate the potential benefits of intraoperative navigation technology.

## 5. Conclusions

The combination of 3D CT-based preoperative planning and intraoperative navigation has proven to be a safe approach, yielding outcomes comparable to standard instrumentation without increasing complication risks. Its advantages include enhanced implant stability through precise placement of fewer but longer screws, optimizing fixation while preserving bone stock—critical for potential future revision surgeries. Additionally, navigation improves screw placement accuracy, reducing risks of suboptimal trajectories and complications such as screw loosening or cortical breaches, without increasing the incidence of neurovascular injuries. This improved mechanical stability may extend prosthetic longevity and enhance long-term patient outcomes.

Given the recent introduction of these technologies, it is not yet possible to present long-term results regarding complication rates or implant longevity. Therefore, further studies are needed to fully elucidate the potential long-term benefits of intraoperative navigation technology.

## Figures and Tables

**Table 1:** Characteristics of the NAV and CONV group regarding demographics, implant features, functional outcomes, and complication rates. The two groups are homogeneous according to male-female ratio and mean age. In bold statistically significant differences.

	NAV	CONV	*p*
**Demographics**			
Gender (male/female)	16/37	6/21	0.24
Age (years); mean ± SD (range)	74.6 ± 6 (58–84)	73.8 ± 5 (62–82)	0.41
Follow-up (months); mean ± SD (range)	30 ± 19 (24–71)	54.5 ± 32 (24–108)	**0.002**
**Intraoperative characteristics**			
Surgical time (min); mean ± SD (range)	98 ± 22 (55–150)	91 ± 22 (55–145)	0.19
Augmented baseplates	36%	25%	0.9
Screws’ number; mean ± SD (range)	2 ± 0.5 (2–5)	2.6 ± 0.8 (2–4)	**0.0047**
Screws length (mm); mean ± SD (range)	36 ± 5 (22–46)	31 ± 4 (18–42)	**<0.00005**
**Functional outcomes** (points); mean ± SD (range)			
Constant Score	66 ± 16 (25–88)	68 ± 16 (32–91)	0.56
Δ Constant Score	10 ± 10 (0–50)	10 ± 9.6 (0–30)	0.96
DASH Score	18 ± 18 (0.8–84.1)	26.4 ± 23 (0.8–71.7)	0.28
**Complications**			
Blood transfusion	7.5%	7.4%	0.18
Others Revision	9.4% 5.6%	11% 11%	1 0.66

## Data Availability

The datasets used and/or analyzed during the current study are available from the corresponding author upon reasonable request.

## List of Abbreviations

3D CT	three-dimensional computed tomography
CONV	conventional group
DASH score	Disability of the Arm, Shoulder, and Hand
GPS^®^	Guided Personalized Surgery
NAV	Navigated group
PJI	periprosthetic joint infection
RSA	reverse shoulder arthroplasty
SD	standard deviation
